# Supplementary material for: Remodelling of the immune landscape by IFNγ counteracts IFNγ-dependent tumour escape in mouse tumour models
Source: Nat Commun. 2025 Jan 2;16:2. doi: 10.1038/s41467-024-54791-0 (PMC11696141; doi:10.1038/s41467-024-54791-0)
Supplement: Supplementary file 1 — Supplementary Information [file 41467_2024_54791_MOESM1_ESM.pdf]

Inventory of Supporting Information

Remodelling of the immune landscapes by IFNγ counteracts IFNγ-dependent tumour escape in mouse tumour models

Vivian W.C. Lau, Gracie J. Mead, Zofia Varyova, Julie M. Mazet, Anagha Krishnan, Edward W. Roberts, Gennaro Prota, Uzi Gileadi, Kim S. Midwood, Vincenzo Cerundolo, Audrey Gérard

Supplementary Figure 1. Relative frequency of mutations in different cancer types.

Supplementary Figure 2: Validation of the B16-OVA IFNγRKO tumour models.

Supplementary Figure 3: Characterisation of the B16-OVA IFNγRKO tumour landscape.

Supplementary Figure 4: Adaptation of Tumour-infiltrating myeloid populations in B16-OVA IFNγRKO tumours.

Supplementary Figure 5: Intra-tumoural function and localisation of myeloid cells.

Supplementary Figure 6: Role of CD8+ T cells for controlling IFNγRKO tumours.

Supplementary Figure 7: Spatial CD8-monocyte crosstalk.

Supplementary Figure 8: Gene signature analysis of 10X Genomics Visium spatial datasets.

Supplementary Table 1. Guide RNA sequences used with CRISPR-Cas9.

Supplementary Table 2. Spectral Flow Cytometry Markers. Antibodies used for spectral flow cytometry.

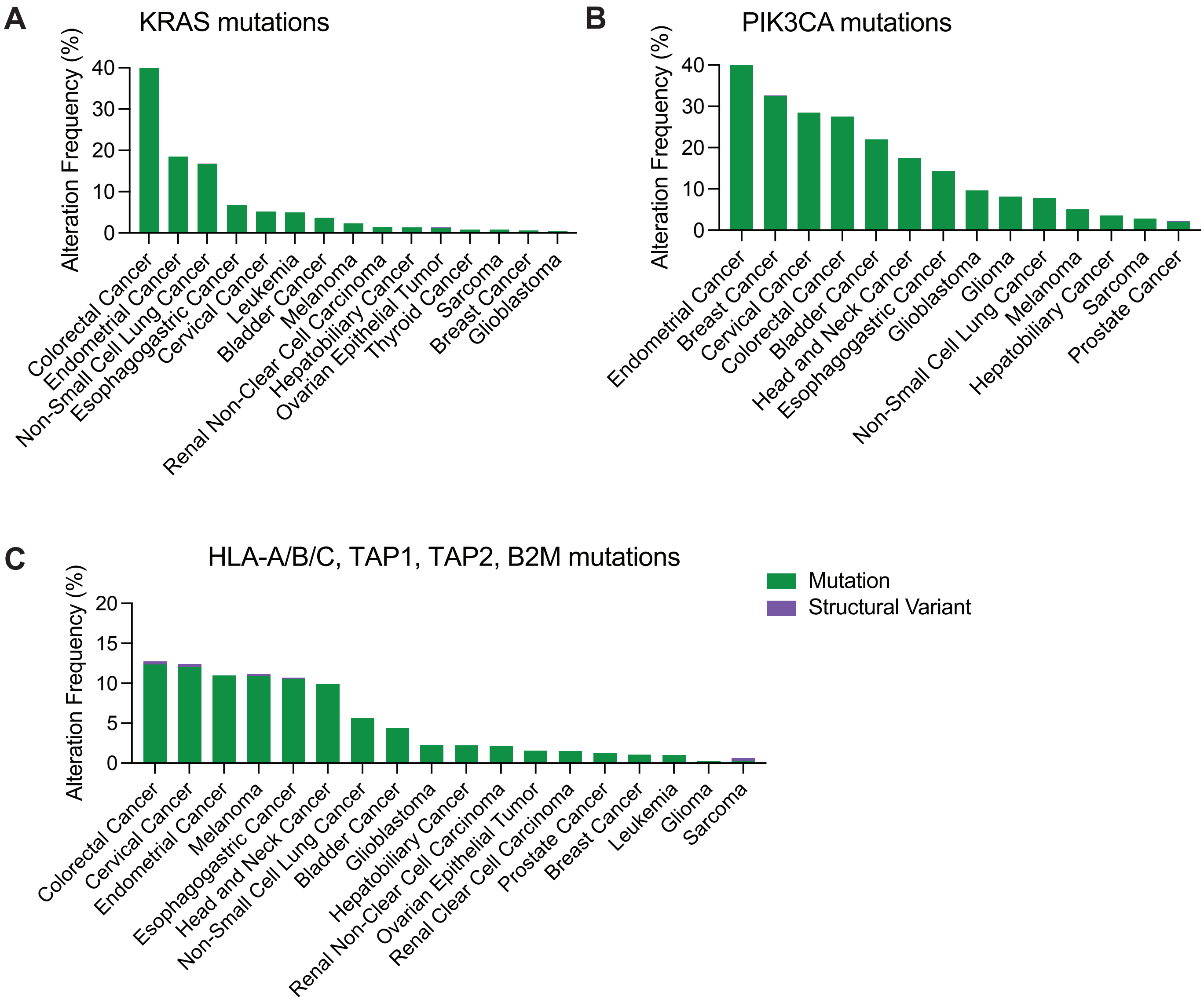

Supplementary Figure 1. Relative frequency of mutations in different cancer types. Relative frequency of KRAS (A), PIK3CA (B), and HLA-pathway (C) mutations in human cancers from cBioPortal, shown as a percentage of all cases in the database. Mutations in genes are shown in green bars and structural variants in purple.

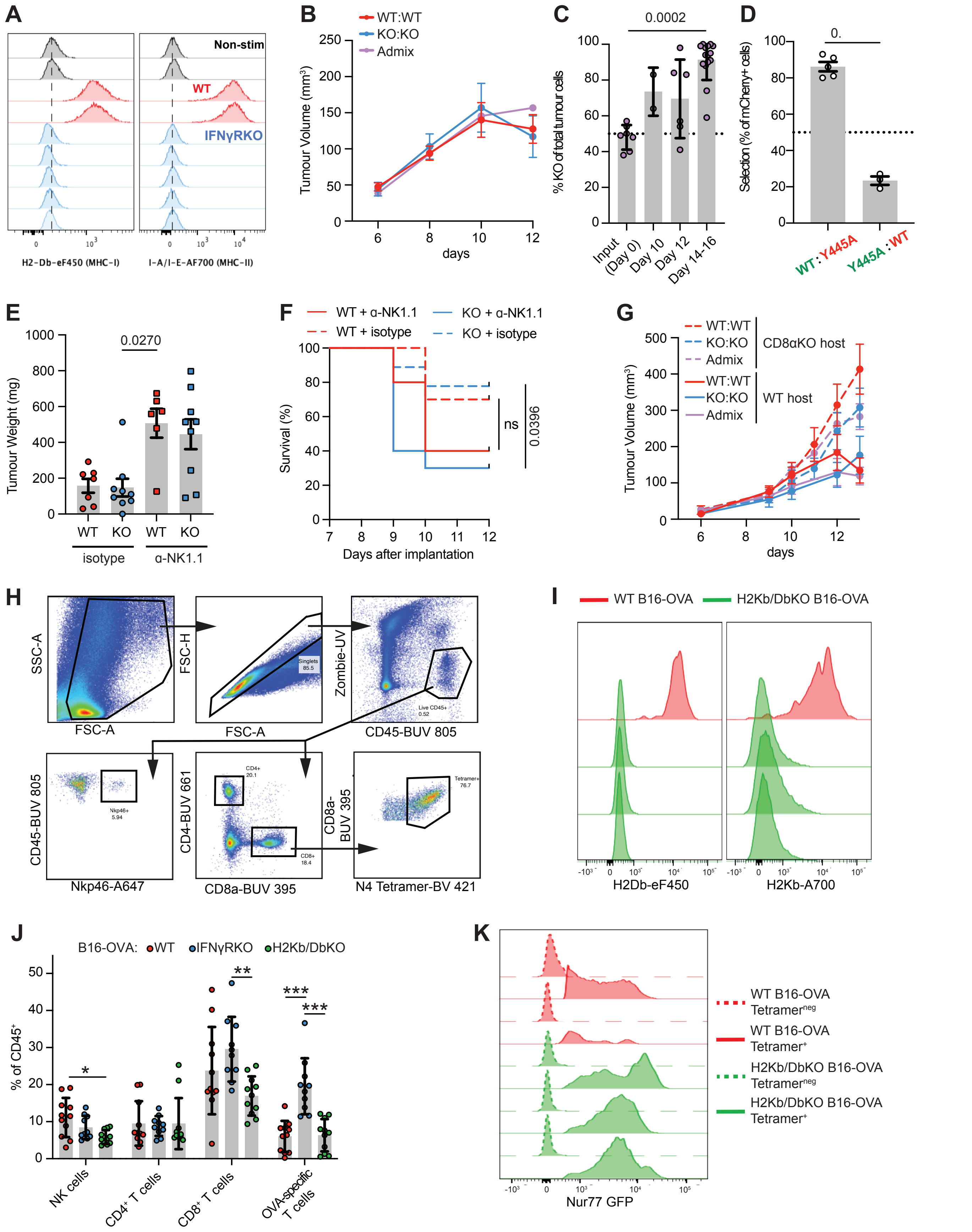

Supplementary Figure 2. Validation of the B16-OVA IFNγRKO tumour models.
(A) In vitro WT (red) and IFNγRKO (blue) B16-OVA cells stimulated with 10ng/mL recombinant murine IFNγ for 48h, and analysed for MHC-I (H2-Db) and MHC-II I-A/I-E expression by flow cytometry. (B) WT (red) and IFNγRKO (blue) tumours expressing mCherry-OVA or ZsGreen-OVA transgenes were admixed and engrafted in WT mice. Tumour volumes of WT (n=10), IFNγRKO (n=10), or admixed (n=18) tumours was quantified over time. Data is a representative of three independent experiments.(C) Percentage of cells from WT/IFNγRKO admixed tumours at indicated time points following tumour engraftment, analysed by flow cytometry. (D) Percentage of cells from tumours composed of B16-OVA WT and IFNγR Y445A mutant cells admixed prior to engraftment. Green and red writing indicates cells express ZsGreen and mCherry, respectively. Tumours were analysed at day 14 post-engraftment. Data is from one experiment (n=8).

Sup Figure 2, continued.
(E-F) WT (red) and IFNγRKO (blue) tumours were engrafted in WT mice treated with isotype control (dashed lines) or NK depleting antibodies (solid lines). (E) Tumour volumes at day 12 post-engraftment. (F) Survival over time. (G) WT and IFNγRKO tumours expressing mCherry-OVA or ZsGreen-OVA were admixed prior to engraftment in WT (solid lines) or CD8αKO (dashed lines) mice. Tumour volumes of WT (red; n[WT mice]=12, n[CD8αKO mice]=11), IFNγRKO (blue; n[WT mice]=8, n[CD8αKO mice]=11), or admixed (purple; n[WT mice]=15, n[CD8αKO mice]=32) tumours was quantified over time. Data is from 2 independent experiments. (H) Gating example for analysis of lymphocyte populations in tumours. (I) mCherry+ B16-OVA WT (red) or H2-Kb/DbKO (green) tumour cells were engrafted in WT mice. Tumours were harvested after 11 days and analysed by flow cytometry. Histogram shows surface expression of MHC-I H2-Db (left) and MHC-I H2-Kb (right) on mCherry+ CD45- cells. (J) B16-OVA WT (red; n=11), IFNγRKO (blue; n=10) or H2-Kb/DbKO (green; n=11) tumour cells were engrafted in WT mice. Tumours were harvested after 11-16 days and analysed by flow cytometry. Tumour-infiltrated lymphocyte populations as a percent of total CD45+ cells were analysed by flow cytometry. Data are from 2 independent experiments. (K) mCherry+ B16-OVA WT (red) or H2-Kb/DbKO (green) tumour cells were engrafted in Nur77-GFP mice. Tumours were harvested after 11 days and analysed by flow cytometry. Histogram shows GFP expression of CD3+ CD8+ tetramer- (dashed lines) or CD3+ CD8+ tetramer+ (solid lines) cells. Data show mean ± SEM with p-values by non-parametric Mann-Whitney t tests for comparisons between two groups, or Kruskal-Wallis tests between three groups with multiple comparisons using Dunn's method.

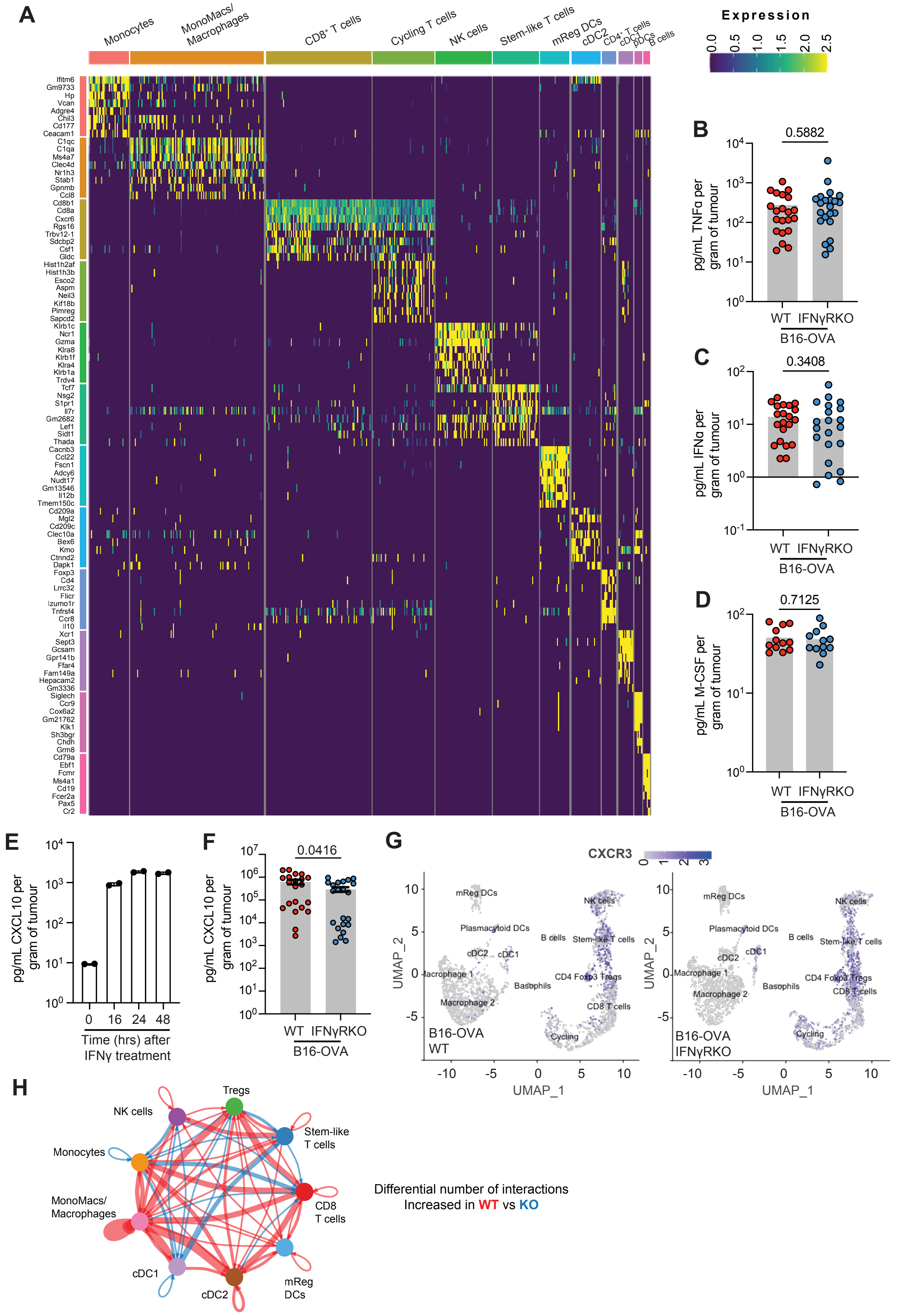

Supplementary Figure 3. Characterisation of the B16-OVA IFNγRKO tumour landscape.
(A) Heatmap of the top 8 genes expressed by unique clusters identified by scRNAseq from Fig.3. (B-D) Quantification of TNF (B n=21), IFNγ (C n=13) and M-CSF (D n=21) concentration from WT (red) and IFNγRKO (blue) tumour supernatant samples by Legendplex multiplex cytokine bead array analysis.

Sup Figure 3, continued.
(E) WT B16-OVA cells were treated with 10ng/ml IFNγ as indicated. Quantification of CXCL10 concentration from tumour supernatant samples by Legendplex multiplex cytokine bead array analysis (n = 2). (F) Quantification of CXCL10 concentration from WT (red) and IFNγRKO (blue) tumour supernatant samples by Legendplex multiplex cytokine bead array analysis (n=21). (G) Feature plots of scRNAseq data from Fig.3 showing relative gene expression of key monocyte/macrophage genes. (H) Circle plot visualizing number of signalling interactions between immune populations from WT and IFNγRKO tumours. Vertices represent independent populations, and arrows indicate direction of signals sent, where broader lines represent increased quantity of signalling interactions. Data show mean ± SEM with p-values by non-parametric Mann-Whitney t tests for comparisons between two groups (B-D, F) or one-way ANOVA with multiple comparisons (E).

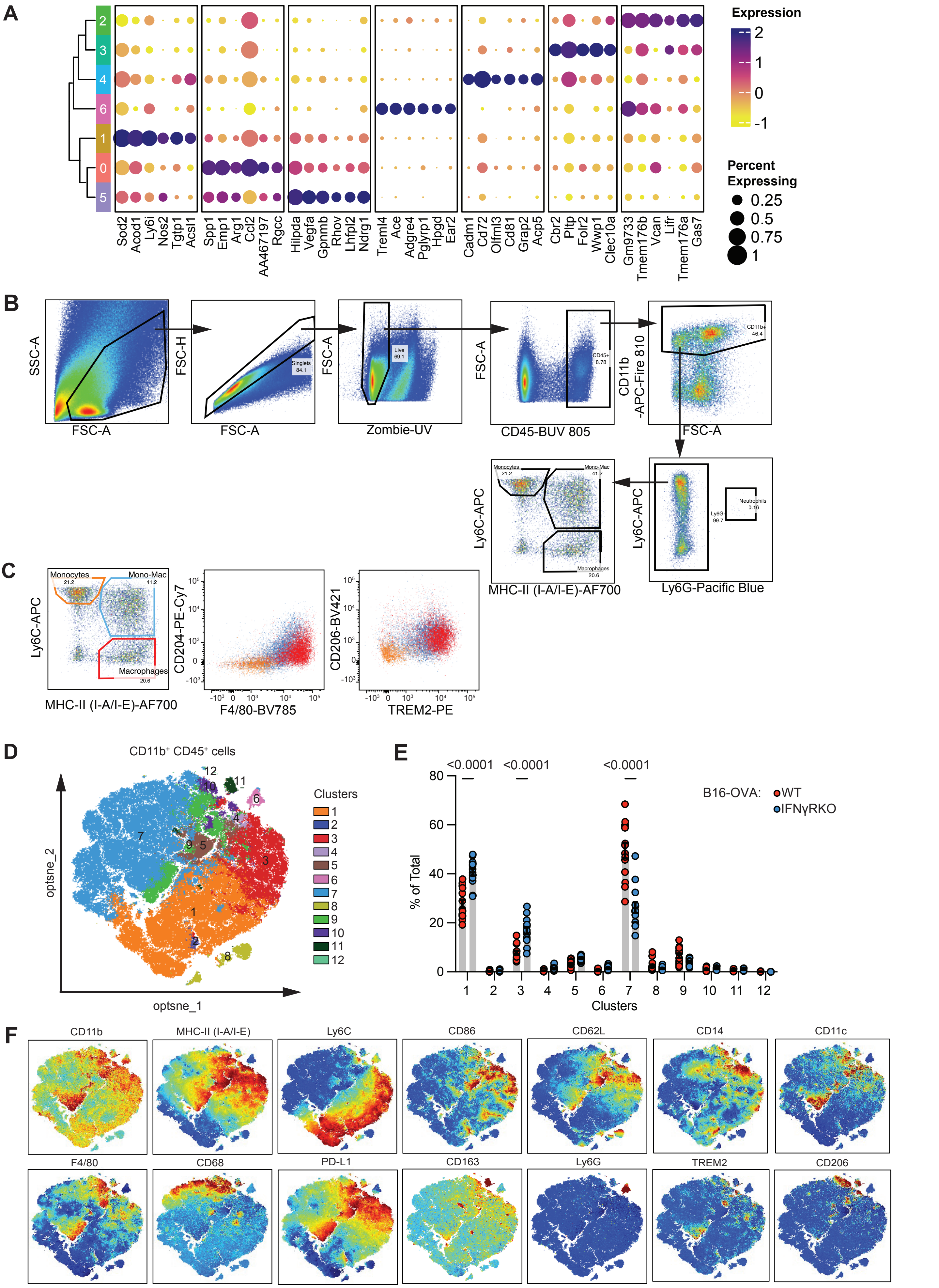

Supplementary Figure 4. Adaptation of Tumour-infiltrating myeloid populations in B16-OVA IFNγRKO tumours.
(A) Clustered dot plot of myeloid subpopulations from scRNAseq with dots coloured by top 6 genes for each cluster, and size of dots representing the percent of cells expression that gene. (B-F) WT or IFNγRKO tumours were engrafted in WT mice and analysed by flow cytometry at endpoint. (B) Gating example for analysis of monocyte and macrophage populations in the tumour. (C) Representative dot plot for Ly6C and MHC-II expression which is gated by monocytes (Ly6C+/MHC-II-), mono-macs (Ly6C+/MHC-II+) and macrophages

Sup Figure 4, continued.
(Ly6C-/MHC-II+), with expression of F4/80, CD204, CD206 and TREM2 shown for each subpopulation by gate colour. (D) optSNE plots of spectral flow cytometry data which shows clusters identified by OMIQ software analysis. (E) Relative quantification of clusters 1-12 of WT (red) and IFNγRKO (blue) tumours. Data show mean ± SEM with p-values by two-way ANOVA tests with multiple comparisons correction using Dunn's method. (F) Pseudocolour plots of individual marker expression, mapped to the optSNE projections. Colours shown (high expression in red, low expression in blue) are respective to each marker. Plots in D-F are from n=8-10 tumours for WT and IFNγRKO from one experiment, and representative of three independent experiments.

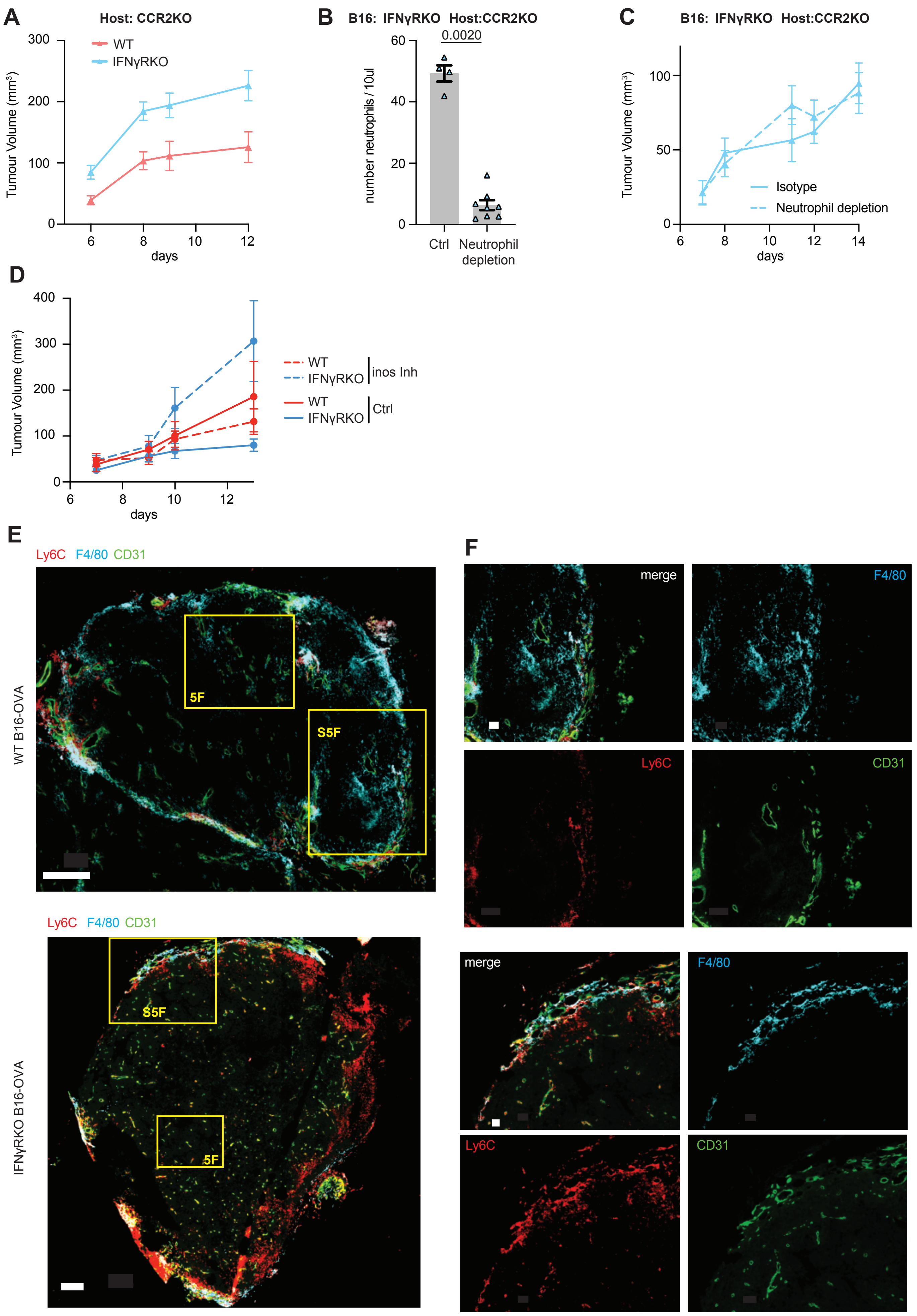

Supplementary Figure 5. Intra-tumoural function and localisation of myeloid cells.
(A) B16-OVA WT (red; n=12) and IFNγRKO (blue; n=14) cells were engrafted in CCR2KO mice. Tumour volumes were quantified over time. Data is representative of 2 independent experiments. (B-C) B16-OVA IFNγRKO cells were engrafted in CCR2KO mice. Mice were treated with depleting neutrophil antibodies when indicated. (B) Efficient intra-tumoural neutrophil depletion was assessed by flow cytometry at endpoint (n[Isotype]=4, n[Neutrophil-depleted]=8). Data show mean ± SEM with p-values by t tests.

Sup Figure 5, continued.

(C) Volumes of Isotype-treated (solid lines; n=10) or neutrophil-depleted (dashed lines; n=12) tumours was quantified over time. Data are from 2 independent experiments. (D) B16-OVA WT (red) and IFNγRKO (blue) cells were engrafted in WT mice. Mice were treated with the INOS inhibitor L-NAME (dashed lines) when indicated. Tumour volumes of WT (n[ctrl]=7, n[L-NAME]=9) or IFNγRKO (n[ctrl]=7, n[L-NAME]=7) tumours was quantified over time. Data is a representative of 2 independent experiments. (E-F) WT or IFNγRKO tumours were engrafted in WT mice and imaged between day 11 to 13. Localisation of Ly6C (red), F4/80 (Cyan) relative to blood vessels (CD31, green) in whole tumour (E) or at the margin (F). Scale bar = 300um (E) and 50um (F). Yellow squares in (E) represent the location from zooms in (F) and Fig.5G.

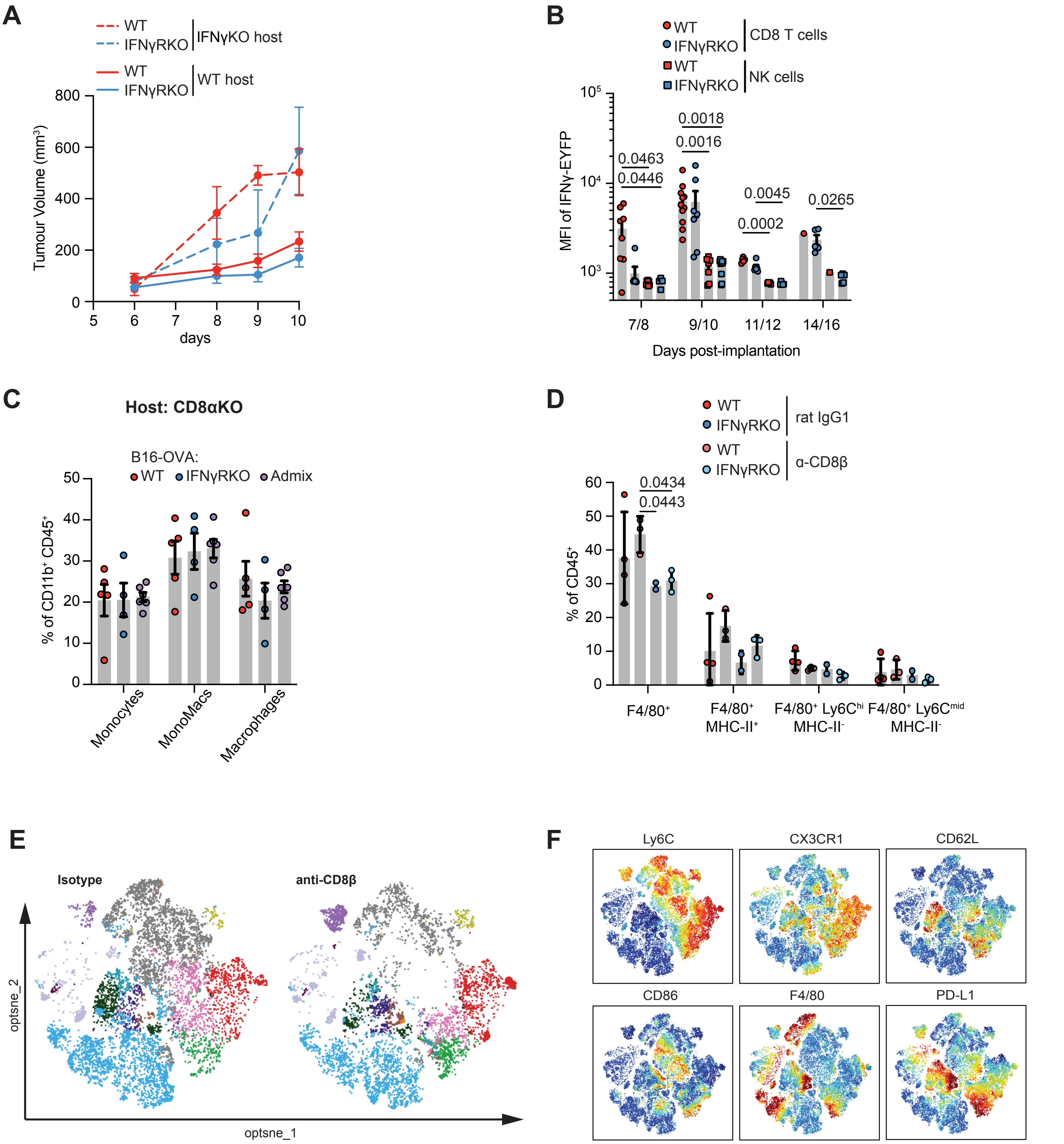

Supplementary Figure 6. Role of CD8+ T cells for controlling IFNγRKO tumours.
(A) B16-OVA WT (red) and IFNγRKO (blue) cells were engrafted in WT (solid lines) or IFNγKO (dashed lines) mice. Tumour volumes of WT (n[WT mice]=7, n[IFNγKO mice]=7) or IFNγRKO (n[WT mice]=7, n[IFNγKO mice]=6) tumours was quantified over time. Data is representative of 3 independent experiments.
(B) WT (red) or IFNγRKO (blue) tumours were engrafted in GREAT mice and harvested when indicated. IFNγ expression was quantified EYFP gMFI in tumour-infiltrating CD8+ T cells and NK cells. Data are pooled from four independent experiments, with timepoints varying between experiments. Data show mean ± SEM with p-values by Kruskal-Wallis tests multiple comparisons correction using Dunn's method.
(C) Infiltration of myeloid populations relative to total CD11b+CD45+ cells in WT (red), IFNγRKO (blue) or admix (purple) tumours engrafted into CD8aKO mice (n[WT]=5, n[IFNγRKO]=4, n[admix]=6).
(D-F) WT or IFNγRKO tumours were engrafted in WT mice, and treated with control (dark) or CD8b-depleting (light) antibodies before and following tumour engraftment. (D) Frequency of specific macrophage (F4/80+) subsets from WT (n[isotype]=4, n[anti-CD8b]=3) or IFNγRKO (n[isotype]=2, n[anti-CD8b]=3) tumours following CD8+ T cell depletion was analysed by flow cytometry 13 days post-engraftment. Data show mean ± SEM with p-values by Kruskal-Wallis tests multiple comparisons correction using Dunn's method.
(E) optSNE plots of high-dimension flow cytometry data of CD11b+ CD45+ tumour infiltrating cells from anti-CD8b and isotype-treated tumour-bearing mice. (F) Pseudocolour overlays of marker expression by different cell clusters. optSNE plots are concatenated data from three biological replicates for each condition. Data is from one experiment.

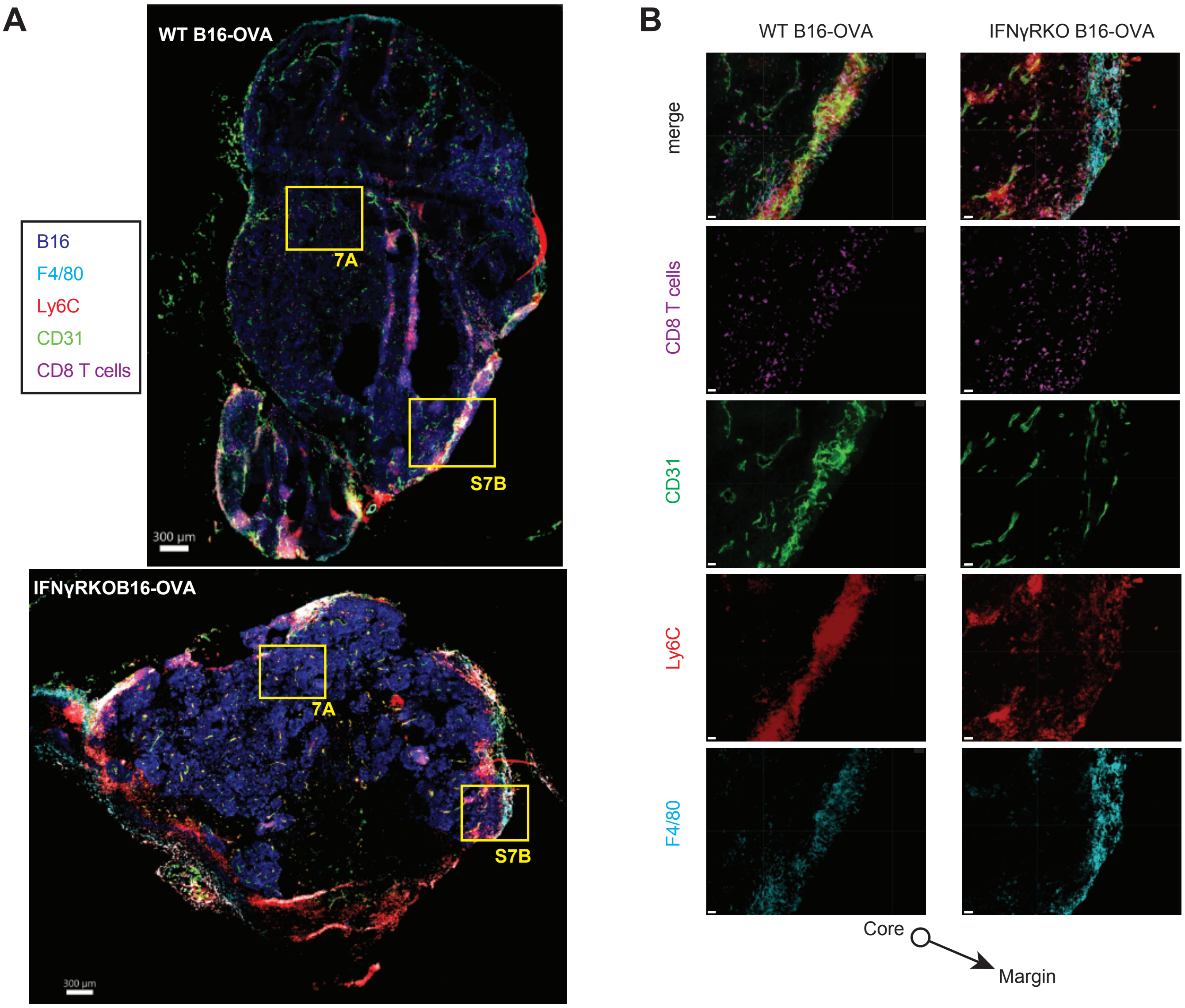

Supplementary Figure 7. Spatial CD8-monocyte crosstalk.
(A-B) WT or IFNγRKO tumours were engrafted in WT mice and imaged between Day 11 to 13. Localisation of Ly6C (red), F4/80 (cyan), and CD8+ T cells (magenta) relative to blood vessels (CD31, green) in whole tumour (A) or at the margin (B). Scale bar = 300um (A) and 30um (B). Yellow squares in (A) represent the location from zooms in (B) and Fig.7A.

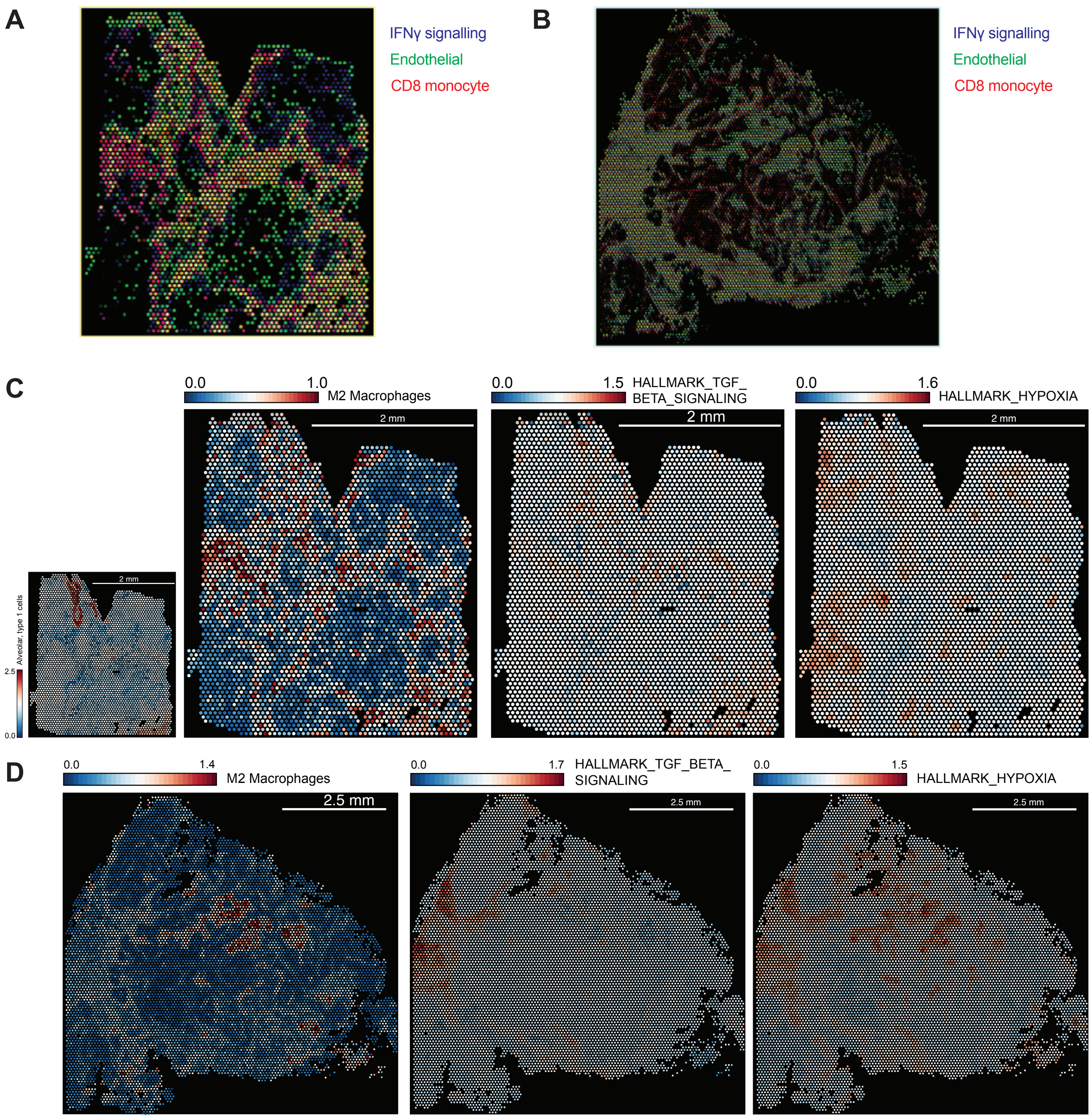

Supplementary Figure 8. Gene signature analysis of 10X Genomics Visium spatial datasets.
(A-B) Overlay of hallmark IFNγ response (blue), CD8-monocyte (red), and endothelial cell (green) gene signatures from 10X Genomics Visium datasets of human lung squamous cell carcinoma (B) or colon adenocarcinoma (C) samples from Fig.7A-B. (C-D) Analysis of 10X Genomics Visium datasets for M2 macrophages, hallmark TGFbeta signalling and hypoxia gene signatures in human lung squamous cell carcinoma (C) or colon adenocarcinoma (D) samples. Gene set expression is indicated by heatmap, where colours represent log-normalized average expression.

Target		Sequence (5' -> 3')	exon	strand	reference
mIFNGR1	fwd<br>rev	CCAC GGTATTCCCAGCATACGACA<br>AAAC TGTCGTATGCTGGGAATACC	2	anti-sense	N/A
mH2Kb/mH2Db	fwd<br>rev	CCAC GCCCCGACTCAGACCCGCGC<br>AAAC GCGCGGGTCTGAGTCGGGGC	1	sense	Tu *et al.* (2017) Cancer Immunol. Res.

Supplementary Table 1.
Guide RNA sequences used with CRISPR-Cas9.

Peak Channel	Fluorochrome	Marker	Clone	Manufacturer	Cat. No.	Dilution (1:X)
UV2	Spark387	CD11c	53-6.7	Biolegend	100798	400
UV6	Zombie UV	--	--	Biolegend	423108	1000
UV9	BUV615	B220	RA3-6B2	eBioscience	366-0452-82	200
UV10	BUV661	CD4	RM4-5	eBioscience	376-0042-82	200
UV14	BUV737	CD44	IM7	eBioscience	367-0441-82	400
UV16	BUV805	CD45	30-F11	eBioscience	368-0451-82	400
V1	BV421	CD206	C068C2	Biolegend	141717	200
V3	PB	Ly6G	1A8	Biolegend	127612	800
V10	BV605	CD86	GL-1	Biolegend	105037	200
V11	BV650	PD-L1	10F.9G2	Biolegend	124333	400
V13	BV711	CD62L	MEL-14	Biolegend	121439	200
V15	BV785	F4/80	BM8	Biolegend	123115	200
B2	ZsGreen	Tumour	GK1.5	Biolegend	124334	400
B10	PerCP-Vio77(	CD68	FA-11	Miltenyi Biotec	130-102-926	20
YG1	PE	TREM2	237920	R&D Systems	FAB17291P	200
YG3	mCherry	Tumour	--	--	--	--
YG9	PE-Cy7	CD163	S15049I	Biolegend	155320	200
R1	APC	Ly6C	HK1.4	Biolegend	128015	400
R2	AF647	NKp46	29A1.4	Biolegend	137628	100
R4	AF700	MHC-II	M5/114.15.2	Biolegend	107621	400
R7	APC/Cy7	CX3CR1	SA011F11	Biolegend	149048	200
R8	APC-Fire810	CD11b	M1/70	Biolegend	101288	400

Supplementary Table 2. Spectral Flow Cytometry Markers.
Antibodies used for spectral flow cytometry.
