# Supplementary material for: Remodelling of the immune landscape by IFNγ counteracts IFNγ-dependent tumour escape in mouse tumour models
Source: Nat Commun. 2025 Jan 2;16:2. doi: 10.1038/s41467-024-54791-0 (PMC11696141; doi:10.1038/s41467-024-54791-0)
Supplement: Supplementary file 2 — Description of Additional Supplementary Files [file 41467_2024_54791_MOESM2_ESM.pdf]

Description of Additional Supplementary Files

Supplementary Data 1. Gene signatures used in the study.
