# Supplementary material for: Remodelling of the immune landscape by IFNγ counteracts IFNγ-dependent tumour escape in mouse tumour models
Source: Nat Commun. 2025 Jan 2;16:2. doi: 10.1038/s41467-024-54791-0 (PMC11696141; doi:10.1038/s41467-024-54791-0)
Supplement: Supplementary file 4 — Reporting Summary [file 41467_2024_54791_MOESM4_ESM.pdf]

nature portfolio

Corresponding author(s): Audrey Gerard
Last updated by author(s): 2024/11//05

Reporting Summary

Nature Portfolio wishes to improve the reproducibility of the work that we publish. This form provides structure for consistency and transparency in reporting. For further information on Nature Portfolio policies, see our Editorial Policies and the Editorial Policy Checklist.

Statistics

For all statistical analyses, confirm that the following items are present in the figure legend, table legend, main text, or Methods section.

n/a	Confirmed	
☐	☒	The exact sample size (*n*) for each experimental group/condition, given as a discrete number and unit of measurement
☐	☒	A statement on whether measurements were taken from distinct samples or whether the same sample was measured repeatedly
☐	☒	The statistical test(s) used AND whether they are one- or two-sided<br>*Only common tests should be described solely by name; describe more complex techniques in the Methods section.*
☒	☐	A description of all covariates tested
☒	☐	A description of any assumptions or corrections, such as tests of normality and adjustment for multiple comparisons
☐	☒	A full description of the statistical parameters including central tendency (e.g. means) or other basic estimates (e.g. regression coefficient) AND variation (e.g. standard deviation) or associated estimates of uncertainty (e.g. confidence intervals)
☐	☒	For null hypothesis testing, the test statistic (e.g. *F*, *t*, *r*) with confidence intervals, effect sizes, degrees of freedom and *P* value noted<br>*Give P values as exact values whenever suitable.*
☒	☐	For Bayesian analysis, information on the choice of priors and Markov chain Monte Carlo settings
☒	☐	For hierarchical and complex designs, identification of the appropriate level for tests and full reporting of outcomes
☒	☐	Estimates of effect sizes (e.g. Cohen's *d*, Pearson's *r*), indicating how they were calculated

Our web collection on statistics for biologists contains articles on many of the points above.

Software and code

Policy information about availability of computer code

Data collection	Flow cytometry data was recorded using BDFACSDiva (v8.0) software<br>No new bioinformatics tools or algorithms were created
Data analysis	Flow cytometry data was analyzed using Flowjo (v10.4.2) or OMIQ (Dotmatics).<br>Single cell RNA sequencing data was analyzed using CellRanger multi (version 6.0.0), Seurat (v4.0.6), UCell (v.1.3), Monocle3 (v.1.0.0), SingleR, CellChat, decontX, SingleR. Bulk data was analysed with consensusTME, ssGSEA<br>Data was visualized using Graphpad (V8.4.1, Prism software), GGplot2(v3.3.5) and ggpubr(v0.5.0).<br>Imaging was analyzed with Imaris (Bitplane – version 10)

For manuscripts utilizing custom algorithms or software that are central to the research but not yet described in published literature, software must be made available to editors and reviewers. We strongly encourage code deposition in a community repository (e.g. GitHub). See the Nature Portfolio guidelines for submitting code & software for further information.

Data

Policy information about availability of data

All manuscripts must include a data availability statement. This statement should provide the following information, where applicable:

- Accession codes, unique identifiers, or web links for publicly available datasets
- A description of any restrictions on data availability
- For clinical datasets or third party data, please ensure that the statement adheres to our policy

The mouse scRNAseq data generated in this study have been deposited in the GEO database under accession code GSE260972. Datasets retrieved from 10X Genomics are licensed under the Creative Commons Attribution license. All data are included in the Supplemental Information or available from the authors upon reasonable requests, as are unique reagents used in this Article. The raw numbers for charts and graphs are available in the Source Data file whenever possible. Source data are provided with this paper.

Research involving human participants, their data, or biological material

Policy information about studies with human participants or human data. See also policy information about sex, gender (identity/presentation), and sexual orientation and race, ethnicity and racism.

Reporting on sex and gender	n/a
Reporting on race, ethnicity, or other socially relevant groupings	n/a
Population characteristics	n/a
Recruitment	n/a
Ethics oversight	n/a

Note that full information on the approval of the study protocol must also be provided in the manuscript.

Field-specific reporting

Please select the one below that is the best fit for your research. If you are not sure, read the appropriate sections before making your selection.

☒ Life sciences ☐ Behavioural & social sciences ☐ Ecological, evolutionary & environmental sciences

For a reference copy of the document with all sections, see nature.com/documents/nr-reporting-summary-flat.pdf

Life sciences study design

All studies must disclose on these points even when the disclosure is negative.

Sample size	Sample size was estimated based on prior experience and complexity. Whenever possible, we used g*power to calculate sample size. Sample size was computed to detect difference of 50% in a treated group with a standard deviation of 20%, false positive of 0.05, and power of 0.80. Sample size was therefore calculated to be between 7-8. Where smaller or bigger changes were expected, sample sizes were increased accordingly.
Data exclusions	no data exclusion
Replication	All findings were replicated in two to four separate experiments (as specified in legends), with the exception of the single cell RNA sequencing experiment, in which data was aggregated from multiple separate biological samples as indicated in the figure legends and methods section.<br>All separate experiments yielded comparable trends and results.
Randomization	For mouse experiments involving multiple genotypes, we used littermates, and controlled that gender and age was similar between all groups, but other than this, allocation was random.<br>Whenever possible, comparison was done between populations within the same mouse, for which randomization is not required.<br>For re-analysis of human data, we used the assignments of clusters from the original study.
Blinding	No blinding was performed during mouse experiments when all mice within an experiment received identical treatments. No subjective scoring methods which would require blinding were used. Most experiments were performed and repeated independently by two researchers.<br>Flow cytometry data were collected in an automatic and unbiased manner.

Reporting for specific materials, systems and methods

We require information from authors about some types of materials, experimental systems and methods used in many studies. Here, indicate whether each material, system or method listed is relevant to your study. If you are not sure if a list item applies to your research, read the appropriate section before selecting a response.

Materials & experimental systems

n/a	Involved in the study
☐	☒ Antibodies
☐	☒ Eukaryotic cell lines
☒	☐ Palaeontology and archaeology
☐	☒ Animals and other organisms
☒	☐ Clinical data
☒	☐ Dual use research of concern
☒	☐ Plants

Methods

n/a	Involved in the study
☒	☐ ChIP-seq
☐	☒ Flow cytometry
☒	☐ MRI-based neuroimaging

Antibodies

Antibodies used

Staining

Conjugate	Antigen	Clone	Comapny	Catalog #	dilution
Spark387	CD11c	53-6.7	Biolegend	100798	400
BUV615	B220	RA3-6B2	eBioscience	366-0452-82	200
BUV661	CD4	RM4-5	eBioscience	376-0042-82	200
BUV737	CD44	IM7	eBioscience	367-0441-82	400
BUV805	CD45	30-F11	eBioscience	368-0451-82	400
BUV395	CD8a	53-6.7	eBioscience	363-0081-82	400
BV421	CD206	C068C2	Biolegend	141717	200
PB	Ly6G	1A8	Biolegend	127612	800
BV605	CD86	GL-1	Biolegend	105037	200
BV650	PD-L1	10F.9G2	Biolegend	124333	400
BV711	CD62L	MEL-14	Biolegend	121439	200
BV785	F4/80	BM8	Biolegend	123115	200
PerCP-Cy5	PD-L1	GK1.5	Biolegend	124334	400
PerCP-Vio770	CD68	FA-11	Miltenyi Biotech	130-102-926	20
PE	TREM2	237920	R&D Systems	FAB17291P	200
PE-Cy7	CD163	S15049I	Biolegend	155320	200
APC	Ly6C	HK1.4	Biolegend	128015	400
AF647	NKp46	29A1.4	Biolegend	137628	100
AF700	MHC-II	M5/114.15.2	Biolegend	107621	400
APC/Cy7	CX3CR1	SA011F11	Biolegend	149048	200
APC-Fire810	CD11b	M1/70	Biolegend	101288	400
AF647	KI67	16A8	Biolegend	652408	200
AF488	GFP	polyclonal	Invitrogen	A-21311	200
eF450	H2Db	28-14-8	Invitrogen	48-5999-80	400
AF700	H2Kb	AF6-88.5	Biolegend	116521	200

Alexa Fluor 647- or BV421-conjugated, N4-specific MHC I tetramers (National Institutes of Health Tetramer Core Facility [Emory University, Atlanta]) 100

in vivo blocking
BioXCell, anti-CD8β Cat. BE0223, and anti-NK1.1 Cat. BE0036
50 μg antibodies every 2 to 3 days
Biolegend, anti-Ly6G (1A8, Cat. 127649) and Thermo Fisher anti-rat Kappa immunoglobulin (MAR18.5, Cat. I-2026)
25ug and 50ug combined antibodies every 2 ays

Validation

All antibodies were obtained from commercial vendors and we based specificity on descriptions and information provided in corresponding Data Sheets available and provided by the Manufacturers.
Dilution optimization was performed on splenocytes or immune cells isolated from tumours.

Biolegend - Flow Cytometry Reagents:-Specificity testing of 1-3 target cell types with either single- or multi-color analysis (including positive and negative cell types).-Once specificity is confirmed, each new lot must perform with similar intensity to the in-date reference lot. Brightness (MFI) is evaluated from both positive and negative populations.-Each lot product is validated by QC testing with a series of titration dilutions. https://www.biolegend.com/en-us/quality/quality-control

ebioscience/invitrogen: Part 1—Target specificity verification
This helps ensure the antibody will bind to the correct target. Our antibodies are being tested using at least one of the following methods to ensure proper functionality in researcher's experiments:
Knockout—expression testing using CRISPR-Cas9 cell models

Knockdown—expression testing using RNAi to knockdown gene of interest
Independent antibody verification (IAV)—measurement of target expression is performed using two differentially raised antibodies recognizing the same protein target
Cell treatment—detecting downstream events following cell treatment
Relative expression—using naturally occurring variable expression to confirm specificity
Neutralization—functional blocking of protein activity by antibody binding
Peptide array—using arrays to test reactivity against known protein modifications
SNAP-ChIP™—using SNAP-ChIP to test reactivity against known protein modifications
Immunoprecipitation-Mass Spectrometry (IP-MS)—testing using immunoprecipitation followed by mass spectrometry to identify antibody targets
Part 2—Functional application validation
These tests help ensure the antibody works in a particular application(s) of interest, which may include
(but are not limited to):
Western blotting
Flow cytometry
ChIP
Immunofluorescence imaging
Immunohistochemistry

Miltenyi: To validate the specificity of an antibody, a suitable counterstaining is performed, which verifies the target population. For this approach, the target gene is knocked out in a suitable cell line using site-specific nucleases and the knockout is confirmed by sequencing of the target locus. The antibody is considered to bind specifically to the intended epitope, if no antibody binding to the knockout cells can be detected. The antibody staining is controlled by fluorescence microscopy as well as flow cytometry. Knockdown of target antigens can be used to validate antibody specificity and their use in flow cytometry applications. In this approach, the target antigen is knocked down using RNA interference or RNAi. The translation of the target RNA is inhibited by transfecting cells with small non-coding RNA oligonucleotides. A comparison between the transfected cells with the control cells reveals the specificity of the tested antibody to its antigen.

R&D: All antibodies are tested for cross-reactivity with closely related molecules using a variety of applications, including direct ELISA, to ensure specificity. These efforts are facilitated by our extensive library of in-house developed antigens.

BioXCell: Advanced Binding Validation utilizes a library of recombinant proteins and bioassay expertise to validate that each lot of applicable InVivoPlusTM antibody binds strongly and specifically to its target antigen.

Eukaryotic cell lines

Policy information about cell lines and Sex and Gender in Research

Cell line source(s)	B16 Tyr-/- expresssing mCherry and Ovalbumin (B16OVA) and B16 expressing minOVA were provided by Ed Roberts, Beatson Institute, University of Glasgow. Those were infected with lentivirus expressing mCherry or ZsGreen, and/or KO for the IFNGR1, H2Db or H2Kb using CRISPR.
Authentication	Cell lines used were not authenticated. OVA expression of B16 melanoma cells has been confirmed using in vitro activation assays with transgenic T cells (OT-I respectively). mCherry and ZsGreen expression in B16 melanoma cells has been confirmed by microscopy. Tyrosine KO (Tyr-/-) inhibits melanin formation and could be confirmed macroscopically by looking at the colour of the B16.
Mycoplasma contamination	Cell lines have been regularly tested for Mycoplasma contamination and tested negative
Commonly misidentified lines (See ICLAC register)	No commonly misidentified cell lines were used in this study.

Animals and other research organisms

Policy information about studies involving animals; ARRIVE guidelines recommended for reporting animal research, and Sex and Gender in Research

Laboratory animals	species: mus musculus,<br>strains: C57bl/6, GREAT, CD8 KO, CCR2 KO, OTI<br>sex: male and female<br>age: 6-14 week-old for in vivo experiments and 8- to 10-week-old for in vitro experiments
Wild animals	wild animals were not used in this study
Reporting on sex	Both genders were used in experiments. Males and Females were equally distributed throughout conditions. We did not initially find any difference between males and females and therefore did not take gender into account for analysis.
Field-collected samples	n/a
Ethics oversight	All experiments involving mice were conducted in agreement with the United Kingdom Animal Scientific Procedures Act of 1986 and

performed in accordance with approved experimental procedures by the Home Office and the Local Ethics Reviews Committee (University of Oxford) under UK project licenses P4BEAEBB5 and PP3609558.

Note that full information on the approval of the study protocol must also be provided in the manuscript.

Plants

Seed stocks	n/a
Novel plant genotypes	n/a
Authentication	n/a

Flow Cytometry

Plots

Confirm that:

- [x] The axis labels state the marker and fluorochrome used (e.g. CD4-FITC).
- [x] The axis scales are clearly visible. Include numbers along axes only for bottom left plot of group (a 'group' is an analysis of identical markers).
- [x] All plots are contour plots with outliers or pseudocolor plots.
- [x] A numerical value for number of cells or percentage (with statistics) is provided.

Methodology

Sample preparation	See method section of manuscript. In some experiments, T cells were isolated from the lymph nodes of 6 to 12 weeks-old mice. In other experiments, tumours were dilacerated using scalpels to obtain <1mm sized pieces and resuspended in R10 supplemented with 1 mg/mL Liberase TL (Roche, Cat. 5401020001) and 10 µg/mL DNase I (Roche, Cat. 11284932001) for enzymatic digestion. Tumour suspensions were incubated at 37°C for 30 minutes before physical dissociation of remaining fragments through 70µm cell strainers to obtain single cell suspensions.
Instrument	FACSAria™ II (BD) was used for sorting and Fortessa X-20 or Aurora for analysis
Software	Data collection: BDFACSDiva (v8.0) software<br>Data analysis: FlowJo V.10 (BD), or OMIQ (Dotmatics).
Cell population abundance	Populations were sorted at >95% purity, determined by flow cytometric analysis of post-sort samples
Gating strategy	For myeloid cells, cells were sorted based on expression of CD45, and Cd11b. Different myeloid populations were then dissected using the markers Ly6C, Ly6G and MHC-II<br>For lymphoid cells, cells were sorted based on expression of CD45. Different lymphoid populations were then dissected using the markers CD3, CD4, CD8 and N4-Tetramer

- [x] Tick this box to confirm that a figure exemplifying the gating strategy is provided in the Supplementary Information.
